# Pneumomediastinum, Pneumopericardium, and Epidural Pneumatosis following Adenotonsillectomy: A Very Rare Complication

**DOI:** 10.1155/2018/4531364

**Published:** 2018-08-19

**Authors:** Feride Fatma Görgülü, Ayşe Selcan Koç, Orhan Görgülü

**Affiliations:** ^1^Radiology Department, Adana City Research and Training Hospital, University of Health Sciences, Adana, Turkey; ^2^Otorhinolaryngology Department, Adana City Research and Training Hospital, University of Health Sciences, Adana, Turkey

## Abstract

Adenotonsillectomy is a common surgical otolaryngology procedure that is associated with several complications, including hemorrhage, odynophagia, damage to teeth, taste disorders, atlantoaxial subluxation, lingual edema, infection, and injury of the carotid artery. Pneumomediastinum, pneumopericardium, and epidural pneumatosis are an extremely unusual condition in children with adenotonsillectomy. Treatment should be conservative in the majority of cases and based on benign self-limiting course of these diseases; early recognition can prevent further complications. The combination of pneumomediastinum with epidural pneumatosis, pneumopericardium, retropharyngeal-prevertebral pneumatosis, axillar-perihumeral pneumatosis, and subcutaneous emphysema is also a very rare condition. We present a unique case with the radiological findings of air in all of these areas in a 6-year-old male child with adenotonsillectomy. The case was unusual in that the patient developed this complication 3 hours later after adenotonsillectomy with severe vomitting. The possible mechanism, the algorithm of treatment, and precautions in such cases will be discussed.

## 1. Case

A 6-year-old male child was admitted to our hospital with his parents for adenotonsillectomy with the history of recurrent tonsillitis and obstructive sleep apnea. Examination revealed grade five enlarged tonsils that were almost meeting at the midline. His adenoids were found to be blocking 90% of his airway in a lateral nasopharyngeal X-ray. Preoperative workup including chest radiography and electrocardiography was normal.

Adenotonsillectomy was performed under general anesthesia with endotracheal intubation. Standard surgical technique was used, and dissection of palatine tonsil was performed meticulously on the subcapsular plane to avoid excessive trauma. Adenoids were removed gently by curettage. Hemostasis was performed by means of careful bipolar electrocoagulation. The surgical procedure proceeded uneventfully with no intraoperative or early postoperative complications. The patient recovered from anesthesia well within 15 minutes.

During the first postoperative hours, there were no episodes of coughing, but the patient had an episode of emesis, one severe vomitting attack. Dyspnea and generalised swelling of his face, neck, and chest wall developed immediately after vomitting at the three-hour postoperative period.

He was conscious and in sitting position because of dyspnea. He did not state an obvious chest pain. But he was uncomfortable and moderately anxious. His vital signs were blood pressure, 110/70 mmHg; pulse rate, 135 beats/minute; respiratory rate, 35 breaths/minute with shallow breathing; temperature, 36.7°C; and oxygen saturation, 98% at room air. A physical examination revealed painless swelling of the face, neck, and chest area with marked crepitus on palpation consistent with cervicofacial subcutaneous emphysema. Emphysema was palpated on anterior chest wall from sternum to axillary regions. Inspection of the oropharynx and nasopharynx did not evidence any bleeding from the operation sides or the presence of any mucosal tear and muscle dehiscence. He had a normal neurologic examination including intact cranial nerves, sensation, and muscle strength.

**Figure 1:**
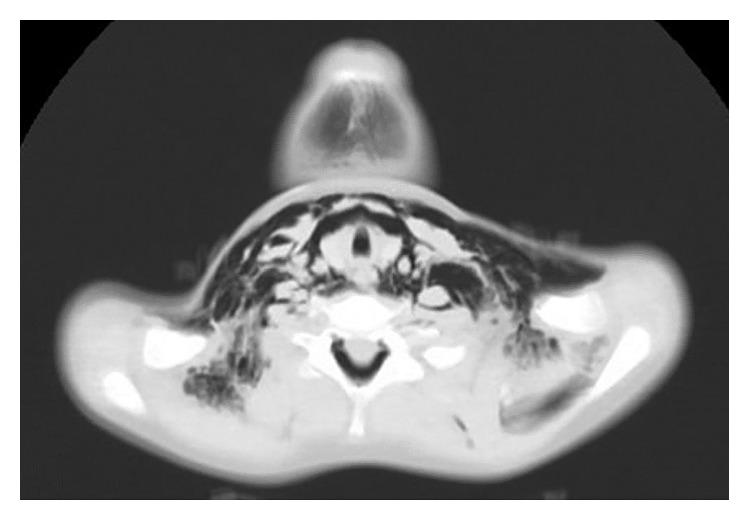
A large volume of emphysema in subcutaneous tissue, the neck planes, peritracheal, and periesophageal spaces.

**Figure 2:**
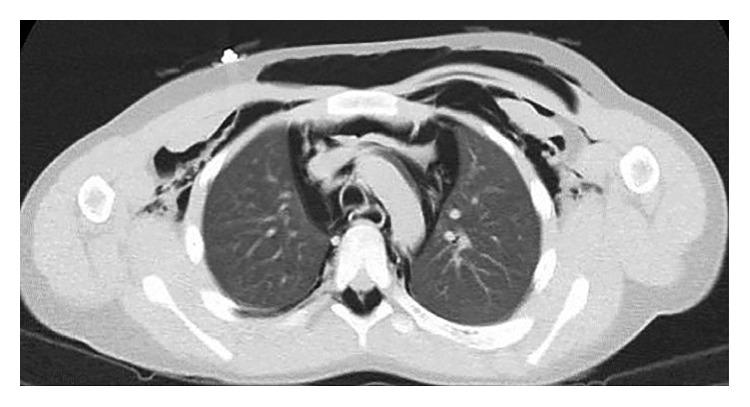
The emphysema of chest wall extended to mediastinum and both axillae.

**Figure 3:**
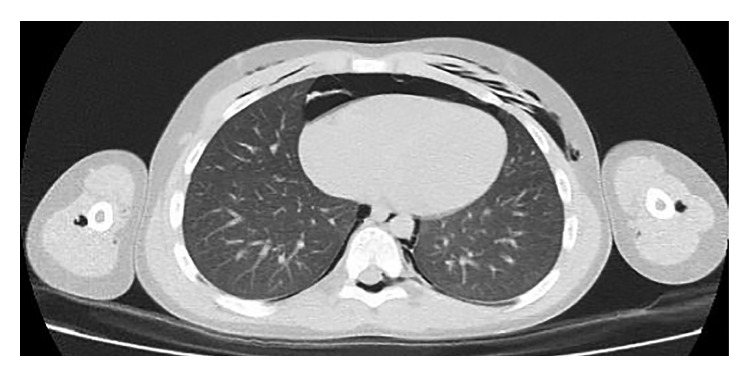
The presence of pneumopericardium, large epidural pneumatosis, air in perihumeral region, and prevertebral pneumatosis without pneumothorax.

**Figure 4:**
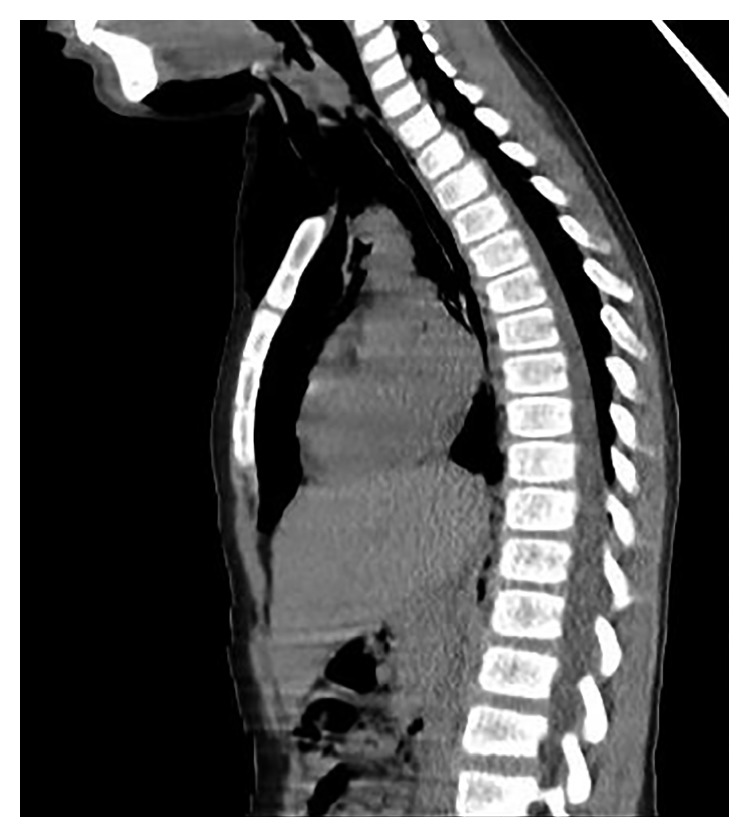
Extensive pneumomediastinum, pneumopericardium, large epidural pneumatosis, and retropharyngeal-prevertebral pneumatosis.

Because of suspicion of subcutaneous emphysema and pneumomediastinum on chest X-ray, a computed tomography (CT) of the thorax was obtained simultaneously. The CT showed the subcutaneous emphysema of the neck and the chest wall along with a large volume of air (Figures 1 and 2). The emphysema extended to both axillae, even the perihumeral region and along the precervical and anterolateral cervical soft tissues (Figure 3). The CT also showed extensive pneumomediastinum, the presence of pneumopericardium, large epidural pneumatosis, retropharyngeal-prevertebral pneumatosis, and air in paraesophageal spaces without pneumothorax (Figure 4). No masses, bullae, and blebs were seen.

Since the patient did not present with additional cardiorespiratory compromise and was hemodynamically stable, conservative management was decided. He was transferred to pediatric intensive care unit for close monitorization and conservative treatment. The patient also was instructed to refrain from coughing. The patient was administered analgesics, oxygen, cold-vapor, and broad-spectrum prophylactic antibiotics, and food intake was forbidden. He was followed up by chest radiographs. He was relieved from signs and symptoms around the sixth day, and his thorax CT revealed absence of air at seventh day after adenotonsillectomy. Then he was discharged at the seventh day, and follow-up examinations did not reveal any abnormal findings.

## 2. Discussion

Subcutaneous emphysema, pneumomediastinum, pneumopericardium, retropharyngeal-prevertebral pneumatosis, and epidural pneumatosis are a quite rare complication of adenotonsillectomy that tends to develop intraoperatively or during the postoperative period [1]. This case is unusual in that these frightening complications developed extensively with severe vomitting at the third hour of postoperative period when there was no other problem.

Pneumomediastinum is the presence of air or another gas within the mediastinum. This is a rare and generally benign self-limited condition that can be categorized as spontaneous or traumatic [2]. Pneumopericardium can be occasionally accompanied with pneumomediastinum and is defined as a collection of air in the pericardial space. If cardiac tamponade develops, mortality increases up to 58% [3]. Epidural pneumatosis secondary to pneumomediastinum is also a benign situation that does not usually cause a neurological deficit. Most cases recover without treatment. However, close follow-up is suggested because of the risk that intracranial infection or paralysis may occur [4].

The mechanism leading to subcutaneous emphysema, pneumomediastinum, and epidural pneumatosis after adenotonsillectomy have not been conclusively identified. There may be two explanations of this mechanism. Firstly, such pneumatic injuries can be caused by surgical trauma where mucosa and muscle are damaged at the surgical site of adenotonsillectomy. In this situation, this injury provides a route for air to enter the parapharyngeal space by following the cervicofascial plane from entering in through the superior constrictor muscle. Because the paraphayrngeal and retropharyngeal spaces are connected anatomically, the air then travels to the mediastinum. The process is facilitated by events such as coughing, vomitting, respiratory infections, and straining that raise upper airway pressure [1, 2, 5]. In the second mechanism, these complications can also arise from injury during intubation, excessive intra-alveolar pressure, and activities that increase the intrathoracic pressure. These may lead to rupture of tracheal mucosa and perivascular alveoli, and then the free air dissects from the ruptured alveoli along the pulmonary vasculature to reach the mediastinum to produce pneumomediastinum. Accumulated mediastinal air may decompress along cervical fascial planes into the subcutaneous tissue to produce subcutaneous emphysema at the chest, head, and neck [6, 7]. Epidural pneumatosis is thought to be due to free air dissecting posteriorly through fascial planes in the nerve roots from the posterior pharynx and mediastinum, axillary arteries, or intervertebral foramina [8]. In our case, there was no obvious mucosal tear and passage of air to the mediastinum in the adenotonsillectomy cavity and upper aerodigestive site. It was more likely that air entered the mediastinum from the lower airways. This event may be a secondary condition to increased intrathoracic pressure with a rupture of the defect in any part of the tracheobronchial tree.

The clinical presentation of subcutaneous emphysema, pneumomediastinum, and pneumopericardium is chest pain, dyspnea, dysphagia, chest and back pain, cyanosis, severe swelling, Hamman's sign (crunching-rasping sound like crepitus synchronous with systole), and crepitus on palpation [9]. Hamman's sign is caused by the heart beating against air-filled tissues, mostly with a decrease in heart sounds, and it is pathognomonic for pneumomediastinum [2]. This sign has also relation with Hamman's syndrome that is a rare condition represented by spontaneous pneumomediastinum and subcutaneous emphysema [10].

Differential diagnosis of neck and chest swollen after adenotonsillectomy includes hematoma, cellulitis, allergic reaction, angioedema, subcutaneous emphysema, and pneumomediastinum [11]. Common predisposing factors for pneumomediastinum were vomiting, coughing, use of cocaine, barotrauma, asthma, excess alcohol consumption, secondary condition to increased intrathoracic pressure, valsalva maneuver, and weight lifting. Uncommon causes of pneumomediastinum include arthroscopy, tooth extraction, vocal training maneuvers, and endotracheal intubation with pyriform sinus, vallecula, or tracheal lacerations [12].

These air containing complications can be detected readily by radiological imaging. If the patient shows the abovementioned clinical conditions, pneumomediastinum should be suspected. A plain neck radiograph will demonstrate the finding of subcutaneous emphysema. Pneumomediastinum should be excluded with an anteroposterior and a lateral chest radiograph [13]. With the increased use of chest CT scan and technologic advancement in the initial evaluation of trauma, the sensitivity of detecting pneumomediastinum has increased, and findings that were not seen on a chest radiograph are increasingly identified. Because the presence of pneumomediastinum posterior to the heart was associated with increased mortality, this location of pneumomediastinum noted on the CT could be immediately assessed on admission. These findings on the CT should alert the clinician that the patient has an increased risk of a mortality of up to 40% and may help guide subsequent management [14]. So the surgeon can consider them for patients as close monitoring in the ICU, aggressive operation, and the possibility of additional diagnostic studies.

In adenotonsil surgery, avoiding harmful maneuvers and using of meticulous bipolar dissection or careful blunt dissection should be performed that delivers less damage, instead of high-voltage electrodissection and aggressive snare technique. This would most probably prevent the development of these frustrating complications. Additionally, as to prevent these complications, intubation should be carefully done to avoid upper airway lesions, and ventilation of high positive oxygen pressure should be avoided in case of significant tissue damage at operative area [2].

Treatment of patients with these complications in children involves intensive cardiorespiratory assessment and monitoring. Any activity that increases upper airway pressure such as coughing, valsalva, or forced expiration should be avoided, and the injured mucosa may be sutured. This provides to prevent further air and microbial tracking within tissue planes. Uncomplicated pneumomediastinum and epidural pneuomatosis are managed conservatively with oxygen, analgesia, sedation, bed rest, restriction of oral intake, the administration of a cough suppressant, and a stool softener. Broad-spectrum antibiotics may also be prescribed to prevent development of life-threatening mediastinitis. In severe cases with airways involvement, orotracheal intubation and tracheotomy are indicated. Surgical approach for decompression is not routinely adopted, if tension pneumothorax, tension pneumomediastinum, air tamponade, and cardiac herniation do not accompany these disorders. Most cases are usually solved after supportive treatment in 7–10 days of observation. The prognosis is excellent with conservative management, and the risk for recurrence is low [15].

## 3. Conclusions

In conclusion, an association between a skin emphysema, a pneumomediastinum, a pneumopericardium, and epidural pneumatosis is extremely rare in children. They are usually benign self-limited conditions that require early differentiation from more serious causes. The defined complications' successfull treatment depends on immediate and accurate diagnosis. Careful examination of initial chest X-ray is important for avoiding unnecessary investigations. CT scanning has an importance in diagnosing clinically suspected cases when chest X-ray is uncertain. In our case, ıt was more likely that air entered through the lower airway to the mediastinum with a rupture anywhere along a defect or bullous formation of the tracheobronchial tree. Intubation should be carefully performed to avoid airway lesions. Ultimately, the surgical approach should be the least aggressive.
